# Quantitative proteomics reveals the importance of nitrogen source to control glucosinolate metabolism in *Arabidopsis thaliana* and *Brassica oleracea*

**DOI:** 10.1093/jxb/erw147

**Published:** 2016-04-16

**Authors:** Daniel Marino, Idoia Ariz, Berta Lasa, Enrique Santamaría, Joaquín Fernández-Irigoyen, Carmen González-Murua, Pedro M. Aparicio Tejo

**Affiliations:** ^1^Department of Plant Biology and Ecology, University of the Basque Country (UPV/EHU), Apdo. 644, E-48080 Bilbao, Spain; ^2^Ikerbasque, Basque Foundation for Science, E-48011 Bilbao, Spain; ^3^Departamento de Ciencias del Medio Natural, Universidad Pública de Navarra, Pamplona, Spain; ^4^Faculdade de Ciências, Centro Ecologia Evolução e Alterações Ambientais, Universidade de Lisboa, Lisboa, Portugal; ^5^Proteomics Unit, Navarrabiomed, Fundación Miguel Servet, Proteored-ISCIII, Instituto de investigación Sanitaria de Navarra (IdiSNA), Pamplona, Spain

**Keywords:** Ammonium, *Arabidopsis*, *thaliana*, broccoli, glucosinolates, myrosinase, nitrate, nitrogen nutrition, proteomics.

## Abstract

Accessing different nitrogen (N) sources involves a profound adaptation of plant metabolism. In this study, a quantitative proteomic approach was used to further understand how the model plant *Arabidopsis thaliana* adjusts to different N sources when grown exclusively under nitrate or ammonium nutrition. Proteome data evidenced that glucosinolate metabolism was differentially regulated by the N source and that both TGG1 and TGG2 myrosinases were more abundant under ammonium nutrition, which is generally considered to be a stressful situation. Moreover, Arabidopsis plants displayed glucosinolate accumulation and induced myrosinase activity under ammonium nutrition. Interestingly, these results were also confirmed in the economically important crop broccoli (*Brassica oleracea* var. *italica*). Moreover, these metabolic changes were correlated in Arabidopsis with the differential expression of genes from the aliphatic glucosinolate metabolic pathway. This study underlines the importance of nitrogen nutrition and the potential of using ammonium as the N source in order to stimulate glucosinolate metabolism, which may have important applications not only in terms of reducing pesticide use, but also for increasing plants’ nutritional value.

## Highlight

A quantitative proteomic approach demonstrates how ammonium nutrition induces glucosinolate biosynthetic and catabolic pathways in Arabidopsis and broccoli.

## Introduction

With the exception of nitrogen-fixing legumes, plants obtain the nitrogen (N) they need for their growth and development from soil. Nitrogen can be found in organic and inorganic compounds; the main available inorganic N forms are nitrate (NO_3_^−^) and ammonium (NH_4_^+^). The proportion of each depends on environmental factors, including the soil’s physical and chemical characteristics (e.g. pH or organic matter content), soil nitrification versus ammonification rates, and soil use. Agricultural soils are often N deficient and need to be supplemented with extra N. Ammonium-based fertilizers formulated with nitrification inhibitors are commonly used to maintain the N available in the soil for longer periods, meaning crops can grow for several weeks with ammonium as the main N source (Huerfano *et al.*, 2015). It is known that the N source influences plant nutrient uptake, cell metabolic homeostasis, and signalling pathways. Indeed, the N source may strongly determine plant response to environmental alterations, both biotic (Gupta *et al.*, 2013) and abiotic (Bloom *et al.*, 2010). All species display a different N source preference; however, assigning this preference is a difficult task because it depends on a complex interaction between genetic, environmental, and ecological factors and there is no robust classification (Britto & Kronzucker, 2002). Although ammonium stress is a general phenomenon, it presents extremely variable inter- and intraspecies thresholds before signs of its impact appear. *Arabidopsis thaliana*, and the Brassicaceae family generally, have been described as being sensitive towards ammonium nutrition (Li *et al.*, 2014; Sarasketa *et al.*, 2014).

Based on physiological studies of the primary metabolism in plants, different strategies have been described to avoid ammonium stress; *inter alia,* NH_4_^+^/NH_3_ extrusion, ammonium compartmentalization, or increasing ammonium assimilation. Launching and maintaining these processes is highly energy-demanding and often results in the most apparent symptom of ammonium toxicity, growth impairment. Ammonium sensitivity can also be mitigated through the modulation of external conditions, for example by controlling external pH, by avoiding low potassium concentrations in the nutrient media, by providing extra carbon/energy, or by supplying minimal amounts of nitrate together with ammonium (Balkos *et al.*, 2010, Hachiya *et al.*, 2012, Sarasketa *et al.*, 2016). In general, the adjustment of primary metabolism towards ammonium nutrition has been studied extensively regarding both carbon and nitrogen metabolism. However, the investigation of the adaptation of secondary metabolism towards ammonium nutrition is still in its infancy. Different studies using Arabidopsis as a model have been useful to find new actors and pathways involved in ammonium stress, including genetic screenings of mutants (Li *et al.*, 2012) and metabolome analysis of cell cultures grown with ammonium (Masakapali et al., 2013), but a proteome study does not yet exist.

Glucosinolates are secondary metabolites derived from amino acids and constitute a group of plant thioglucosides (β-thioglucoside-N hydroxysulfates) mainly found in the Brassicaceae family. There is great diversity in their structure; more than 140 different glucosinolates have been documented and there are at least 30 different glucosinolates in *A*. *thaliana* (Wittstock and Burow, 2010). At present, their function is mainly associated with the products of their degradation. Glucosinolates are degraded by specialized β-thioglucoside hydrolases (TGGs, also called myrosinases) which release isothiocyanates that can then be converted into alternative products, such as thiocyanates, nitriles, or epithionitriles (Halkier and Gershenzon, 2006; Wittstock and Burow, 2010). The most extensively studied function of glucosinolates is related to the ability of cruciferous plants to defend against pathogen attack. Tissue damage, triggered for example by herbivores chewing, causes glucosinolates to breakdown into their degradation products, some of which have insect-deterring functions. More recently, their role in plant defence against microbial pathogens has also been described (Bednarek *et al.*, 2009; Clay *et al.*, 2009). Although less studied, glucosinolates also have an influence on other processes, such as K^+^ channel regulation (Zhao *et al.*, 2008) and salt-stress response (López-Berenguer *et al.*, 2008). Similarly, it has been suggested that they serve as a nutrient sink; for example, they may act as a sulfur sink, given that sulfur deprivation promotes glucosinolate degradation to assist in primary metabolism functions like protein synthesis (Falk *et al.*, 2007).

In this work, we aimed to better understand how plants adapt to different nitrogen sources and to increase our knowledge of how plants respond to ammonium nutrition. To this end, we grew *A. thaliana* plants in axenic hydroponic conditions with either nitrate or ammonium as the exclusive N source, and performed MS quantitative proteomics analysis. Finally, we determined whether the results found in the model plant Arabidopsis could be translated to an economically important Brassica crop, broccoli (*Brassica oleracea* var. *italica*).

## Materials and methods

### Experimental design and growth conditions

*A*. *thaliana* Col-0 seeds were sterilized and sown in Petri dishes with a modified Murashige and Skoog solution (2.25mM CaCl_2_, 1.25mM KH_2_PO_4_, 0.75mM MgSO_4_, 5mM KCl, 0.085mM Na_2_EDTA, 5 µM KI, 0.1 µM CuSO_4_, 100 µM MnSO_4_, 100 µM H_3_BO_3_, 0.1 µM CoCl_2_, 100 µM FeSO_4_, 30 µM ZnSO_4_, and 0.1 µM Na_2_MoO_4_; 0.5% sucrose, 20.5mM MES, pH 6.7, and 0.6% agar) containing 2mM nitrogen in the form of 1mM (NH_4_)_2_SO_4_ for ammonium-based nutrition or 1mM Ca(NO_3_)_2_ for nitric nutrition. To properly compare both N nutrition types, NH_4_^+^-fed plants were supplemented with 1mM CaSO_4_ to compensate for the Ca^2+^ supplied together with the NO_3_^−^. For Supplementary Fig. S1, in order to avoid any potential effect of unbalancing sulfate content, plants were grown with equal amounts of sulfate, comparing 1mM (NH_4_)_2_SO_4_ with 1mM Ca(NO_3_)_2_ supplemented with 1mM CaSO_4_, or alternatively applying NH_4_Cl as the ammonium source.

Plates were kept for 4 days in the dark at 4ºC and then moved into a phytotron with the following controlled conditions: 14h, 200 µmol m^−2^ s^−1^ light intensity, 60% relative humidity, and 22ºC day conditions; and 10h, 70% relative humidity, and 18ºC night conditions. Nine-day-old seedlings were transferred to 24-well plates containing 1mL of the same nutrient solution used for seed germination without agar (one plant per well). Plates were kept under continuous shaking (120rpm) for 12 days. The nutrient solution was renewed on days 5 and 9. Sterility was maintained until harvest. Six independent experiments were performed. In each experiment, 100 plants per nutrition type were harvested and pooled together. When harvesting, shoots and roots were dried with paper towels, the biomass of individual plants recorded, and plants immediately frozen in liquid nitrogen and stored at −80ºC.

Broccoli plants (*Brassica oleracea* L. var *italica*, genotype Monaco, Syngenta) germinated and grown in peat for 3 weeks were transplanted in 1L pots (one plant per pot) with a perlite and vermiculite (1:2) mixture and maintained for 5 weeks in a growth chamber with the following controlled conditions: 14h, 350 µmol m^−2^ s^−1^ light intensity, 60% relative humidity, 22ºC day conditions; and 10h, 70% relative humidity, 18ºC night conditions. Plants were irrigated with nutrient solution (1.15mM K_2_HPO_4_, 2.68mM KCl, 0.7mM CaSO_4_, 0.07mM Na_2_Fe–EDTA, 0.85mM MgSO_4_, 0.5mM CaCO_3_, 16.5 µM Na_2_MoO_4_, 3.7 µM FeCl_3_, 3.4 µM ZnSO_4_, 16 µM H_3_BO_3_, 0.5 µM MnSO_4_, 0.1 µM CuSO_4_, 0.2 µM AlCl_3_, 0.1 µM NiCl_2_, 0.06 µM KI, pH 6.8) exclusively under ammonium (5mM NH_4_Cl) or nitrate nutrition [2.5mM Ca(NO_3_)_2_]. When harvesting, the fresh weight was recorded, and leaves were immediately frozen in liquid nitrogen and stored at −80ºC for subsequent analysis.

### Metabolite determination

Ammonium accumulation in leaves was determined by the phenol hypochlorite assay as described in Sarasketa *et al.* (2014). Nitrate and sulfate content were determined by capillary electrophoresis, using Agilent G1600 CE3D (Agilent Technologies, Santa Clara, CA, USA). The content of chlorophyll a and b and that of anthocyanin was determined using spectrophotometry. For chlorophyll quantification, leaves were extracted in 80% aqueous acetone and the absorbance measured at A_645_ and A_663_ (Arnon, 1949). For anthocyanins analysis, leaves were extracted in 1mL of 3M HCl:H_2_O:MeOH (1:3:16 by volume) and anthocyanin content estimated at A_530_–0.24.A_653_ (Gould *et al.*, 2000). Met and Trp content was determined by high-performance capillary electrophoresis using a Beckman Coulter PA-800 apparatus (Beckman Coulter Inc., Brea, CA, USA) equipped with a fused silica capillary (diameter: 50 μm; length: 43/53.2cm), in an electrophoresis buffer containing 50mM borax and 45mM *α*-cyclodextrin, pH 9.2. Analyses were carried out at 30kV and 20ºC. For this, 50mg of leaves were ground with liquid N_2_ and homogenized with 1M HCl. The resulting mixture was allowed to settle for 10min in ice and centrifuged at 21 000*g* for 10min at 4°C. The supernatants were neutralized and diluted (1:5) with 20mM borate buffer, pH 10, and derivatized before detection with 1mM of fluorescein isothiocyanate in acetone.

For glucosinolate determination, around 100mg of freeze-dried leaf powder was extracted in 1.5mL of 70% MeOH for 30min at 70°C, with vortexing every 5min. Homogenates were then centrifuged (20min, 10 000*g*, 4°C), supernatants collected, and the methanol removed using a rotary evaporator. Finally, the dried residue was reconstituted in 1mL ultrapure water and filtered (0.2 μm inorganic membrane filter). Each sample was analysed in a Waters HPLC system (Waters Cromatografía S.A., Barcelona, Spain), consisting of a W600E multi-solvent delivery system, in-line degasser, W717plus autosampler, and W2996 PAD. The compounds were separated in a Luna C18 column (25×0.46cm, 5 μm particle size; Phenomenex, Macclesfield, UK) with a security guard C18-ODS (4×30mm) cartridge system (Phenomenex). The mobile phase was a mixture of water and trifluoroacetic acid (99.9:0.1, v/v; A) or acetonitrile and trifluoroacetic acid (99.9:0.1, v/v; B). The glucosinolates were eluted off the column in 35min with a flow rate of 1mL/min. After 5min with 1% B, they were separated using a linear gradient reaching 17% B in 20min, 25% B at 22min, 35% B at 30min, 50% B at 35min, and 99% B at 40min. Glucosinolates present in the samples were then identified using a previously described LC-MS method in the Metabolomics Platform of CEBAS-CSIC in Murcia, Spain (Domínguez-Perles *et al.*, 2010) and quantified using sinigrin as the standard at 227nm.

### Myrosinase activity

Myrosinase activity was assayed as described by Barth and Jander (2006). Briefly, 30mg of frozen leaves were ground with 5 × extraction buffer (w/v) [33mM sodium phosphate, pH 7, 5% polyvinylpolypyrrolidone (PVPP), 1mM phenylmethylsulfonyl fluoride (PMSF), 1mM ε-aminocaproic acid, 10 μM leupeptin]. Next, 50 µL of extract (diluted 1:25) was incubated with 200 µL of reaction buffer (33mM sodium phosphate, pH 7, 0.35mM sinigrin, 0.33mM ascorbic acid). Spectrophotometry was used to monitor sinigrin disappearance at 227nm (25°C, 15min).

### Immunoelectrophoresis

For β-thioglucoside glucohydrolase 1 (TGG1; myrosinase 1) and β-thioglucoside glucohydrolase 2 (TGG2; myrosinase 2) content quantification, proteins were extracted from 20mg of leaf powder with 0.4mL of extraction buffer (10mM MgCl_2_, 1mM EDTA, 1mM EGTA, 10mM DTT, 0.1% Triton X-100, 10% glycerol, 0.05% BSA, 0.5% PVPP, 50mM HEPES, pH 7.5) in the presence of a cocktail of proteases inhibitors (1mM PMSF, 1mM ε-aminocaproic acid, 10 μM leupeptin). Samples were then centrifuged at 4000*g* for 30min at 4°C and the supernatants recovered. The protein content of the supernatants was quantified by a dye-binding protein assay (Bio-Rad Bradford Protein assay) with BSA as the standard for the calibration curve. Equal amounts of proteins were loaded onto a 1.5 mm-thick denaturizing 4.6% (w/v) stacking and 10% (w/v) resolving gel. Gels were electroblotted onto a nitrocellulose membrane and blots blocked in 5% (w/v) skim milk in 20mM Tris-buffer saline at 4°C for 1h, washed, and incubated with α-TGG1 or α-TGG2 in a dilution of 1:5000 (Ueda *et al.*, 2006). They were then incubated with goat antirabbit horseradish peroxidase conjugate secondary antibody (1:20 000). Finally, immunoreactive bands were visualized with a Molecular Imager ChemiDoc XRS System (Bio-Rad) and quantified with ImageJ software.

### Sample preparation and labelling for proteomic analysis

Fifty milligrams of leaves were ground in liquid nitrogen and homogenized in 0.5mL extraction buffer [7M urea, 2M thiourea, 4% CHAPS, 2% Triton X-100, 50mM DTT, and 0.5% plant protease inhibitor and phosphatase inhibitors cocktails (Sigma-Aldrich)]. Samples were centrifuged for 15min (10 000*g*, 4°C) and total protein precipitated from 200 µL of supernatant with methanol and chloroform (600 µL methanol, 15 µL chloroform, and 450 µL ultrapure water). Mixtures were vortexed and centrifuged for 1min at 14 000*g*. The aqueous phase was then removed, an additional 450 µL of methanol added, and centrifugation repeated. The methanol phase was removed and the protein pellets dried in a vacuum centrifuge and finally resuspended in a solution containing 7M urea, 2M thiourea, and 4% CHAPS (15 µL). Protein quantification was performed with a dye-binding Bradford micro-assay (Bio-Rad), and a shotgun comparative proteome-wide analysis of total leaf extracts (four biological replicates) was carried out using isobaric tags for relative and absolute quantitation (iTRAQ; Unwin *et al.*, 2010). iTRAQ labelling was performed according to the manufacturer’s protocol (Sciex). Briefly, 100 μg of total protein was reduced with 50mM Tris(2-carboxyethyl)phosphine at 60°C for 1h, and cysteine residues were alkylated with 200mM methylmethanethiosulfonate (MMTS) at room temperature for 15min. Protein enzymatic cleavage was carried out with trypsin (Promega; 1:20, w:w) at 37°C for 16h. An iTRAQ 8-plex experiment was performed labelling each tryptic digest with one isobaric amine-reactive tag according to the manufacturer’s instructions, as follows: Tag113, Ammonium Sample 1; Tag114, Ammonium Sample 2; Tag115, Ammonium Sample 3; Tag116, Ammonium Sample 4; Tag117, Nitrate Sample 1; Tag118, Nitrate Sample 2; Tag119, Nitrate Sample 3; Tag121 Nitrate Sample 4. After 1h incubation, samples were pooled and evaporated to <40 μL in a vacuum centrifuge.

### Peptide fractionation and triple-TOF 5600 MS analysis

To increase the proteome coverage, the peptide pool was injected into an Ettan LC system with a XTerra RP18 pre-column (2.1×20mm) and a high pH stable XTerra RP18 column (C18; 2.1×150mm; 3.5 μm; Waters) at a flow rate of 40 μL/min. Peptides were eluted with a mobile phase B with a 5–65% linear gradient over 35min (A, 5mM ammonium bicarbonate in water at pH 9.8; B, 5mM ammonium bicarbonate in acetonitrile at pH 9.8). In total, 11 fractions were collected, evaporated under vacuum, and reconstituted into 20 μL of 2% acetonitrile, 0.1% formic acid, and 98% ultrapure water prior to MS analysis.

Peptide mixtures were separated by reverse-phase chromatography using an Eksigent NanoLC-Ultra 2D pump fitted with a 75 μm ID column (Eksigent 0.075×25cm). Samples were first loaded for desalting and concentration into a 0.5 cm-length 300 μm ID pre-column packed with the same chemistry as the separating column. Mobile phases were 100% water and 0.1% formic acid (FA) (buffer A), and 99.9% acetonitrile and 0.1% FA (buffer B). The column gradient was developed in a 237min two-step gradient from 5% B to 25% B in 180min and 25% B to 40% B in 30min. The column was equilibrated in 95% B for 10min and 5% B for 15min. During the whole process, the pre-column was in-line with the column and flow maintained all along the gradient at 300 nL/min. Eluting peptides from the column were analysed using a Sciex 5600 TripleTOF™ system. Data were acquired upon a survey scan performed in a mass range of 350 m/z up to 1250 m/z in a scan time of 250ms. The top 35 peaks were selected for fragmentation. The minimum accumulation time for MS/MS was set to 100ms, giving a total cycle time of 3.8s. Product ions were scanned in a mass range of 100 m/z up to 1700 m/z, and excluded for further fragmentation for 15s.

### Proteomics data analysis

Analyses of raw data (.wiff, Sciex) were performed with MaxQuant software (Cox and Mann, 2008). For peak list generation, default Sciex Q-TOF instrument parameters were used except for the main search peptide tolerance, which was set to 0.01Da, and the MS/MS match tolerance, which was increased up to 50ppm. The minimum peptide length was set to six amino acids. Two databases were used. A contaminant database (.fasta) was first used to filter out contaminants. Peak lists were searched against the TAIR10 *A*. *thaliana* database, and Andromeda was used as a search engine (Cox *et al.*, 2011). Methionine oxidation was set as a variable modification, and cysteine modification by MMTS was set as a fixed modification. The maximum false discovery rates (FDR) were set to 0.01 at the protein and peptide levels. Analyses were limited to peptides of six or more amino acids in length, and considering a maximum of two missed cleavages. The relative protein abundance output data files were managed using R scripts for subsequent statistical analyses and representation. Proteins identified by site (identification based only on a modification), reverse proteins (identified by decoy database), and potential contaminants were filtered out. Only proteins with more than one identified peptide were used for quantification. For possible quantification data rescue, up to one missing value for each group was rescued by replacing it with the mean of the rest of the in-group samples. Data were normalized and transformed for later comparison using quantile normalization and log2 transformation, respectively. The Limma Bioconductor software package in R was used for ANOVA analyses. Significant and differential data were selected by a *P* value <0.05, and a fold change <0.64 or >1.5 on a linear scale.

### RNA extraction and quantitative RT-PCR analysis

RNA was extracted from 50mg of leaves (Nucleospin RNA plant kit; Macherey-Nagel) and 1 μg of RNA was retrotranscribed into cDNA (PrimeScript^TM^ RT; Takara Bio Inc.). Gene expression was determined from 2 µL of cDNA diluted 1:10 in a 15 μL reaction volume using SYBR Premix ExTaq^TM^ (Takara Bio Inc.) in a Step One Plus Real Time PCR System (Applied Biosystems). The PCR programme used was as follows: polymerase activation (95°C for 5min), amplification and quantification cycles repeated 40 times (94°C for 15s, 60°C for 1min), and melting curve (40–95°C with one fluorescence read every 0.3°C). Relative expression was calculated as the ∆Cp between each gene and the average of the housekeeping genes *SAND family* (At2g28390) and *β-tubulin 4* (At5g44340) with the primers described in Marino *et al.* (2013). The primers used for genes related to glucosinolate metabolism are described in Li and Sack (2014) and Guo *et al.* (2013a).

## Results

### Arabidopsis physiology under a mild ammonium stress

In a previous study we grew *A*. *thaliana* plants under four different degrees of ammonium stress and observed that an external medium pH of 6.7 helped to alleviate ammonium stress (Sarasketa *et al.*, 2016). In the present work, we chose a pH of 6.7 and a 2mM ammonium concentration to further investigate Arabidopsis behaviour under a mild ammonium stress. In Sarasketa *et al.* (2016), plants were germinated and grown for 9 days prior to treatment onset with 0.5mM ammonium nitrate as the N source at a pH 5.9. However, in the present work, ammonium-fed plants were grown throughout the whole experiment without nitrate in the medium.

Arabidopsis plants accumulated a similar biomass under both nutrition types (Table 1). However, shoot biomass was 10% lower and root biomass was 20% higher under ammonium nutrition, which meant the shoot to root ratio was greater in nitrate-fed plants (Table 1). As expected, the nitrate level was higher in nitrate-fed plants. In contrast, no ammonium, sulfate, or protein accumulated under ammonium nutrition. Chlorophyll accumulation (Sanchez-Zabala *et al.*, 2015) or chlorosis (Li *et al.*, 2012) have been shown to be markers of different ammonium stress degrees. In this work, the chlorophyll content was unaltered. However, the anthocyanin content was higher under ammonium nutrition, indicating that while the plants were generally tolerant towards the imposed treatment, they were actually facing a stressful situation (Table 1).

**Table 1.** Growth parameters, chlorophyll, anthocyanin, ammonium, nitrate, sulfate, and protein content in *Arabidopsis thaliana* plants grown with nitrate or ammonium as nitrogen source

	Nitrate	Ammonium
Whole plant biomass (mg FW)	24.12±0.48	23.56±0.41
Shoot biomass (mg FW·plant^−1^)	17.21±0.39*	15.42±0.44
Root biomass (mg FW·plant^−1^)	6.91±0.25*	8.29±0.46
Shoot to root ratio	2.49±0.12*	1.86±0.08
Chlorophyll a (µg·mg FW^−1^)	0.21±0.01	0.18±0.02
Chlorophyll b (µg·mg FW^−1^)	0.08±0.01	0.07±0.01
Chlorophyll a + b (µg·mg FW^−1^)	0.29±0.02	0.27±0.04
Anthocyanin (nmol·g FW^−1^)	0.08±0.01*	0.26±0.02
NH_4_^+^ (nmol·mg FW^−1^)	0.17±0.01	0.17±0.01
NO_3_^−^ (nmol·mg FW^−1^)	2.68±0.37*	0.25±0.07
SO_4_^−2^ (µmol·mg FW^−1^)	38.29±17.17	36.76±1.34
Protein (µg·mg FW^−1^)	3.94±0.11	4.36±0.23

Values represent mean±SE (for growth parameters *n* = 35, for metabolic parameters *n* = 6). Statistical differences according to a Student’s *t*-test *P* value < 0.05 are indicated by an asterisk. FW, fresh weight.

### Nitrogen source modulated Arabidopsis proteome

To further understand how plants respond to long-term growth under ammonium nutrition, we carried out a quantitative proteome-wide study. To do so, we performed an iTRAQ 8-plex experiment, analysing four samples per treatment, with each sample corresponding to a pool of 100 plants. We identified 3760 proteins and, following the criteria described in the ‘Materials and methods’, we quantified 2108 proteins (Supplementary Dataset S1 contains the complete list of proteins identified, quantified, and differentially expressed; Supplementary Dataset S2 provides the information about all the peptides identified). Out of the quantified proteins, 144 were differentially expressed (fold-change ratios ≥ 1.5; *P <* 0.05), 75 were more abundant under ammonium nutrition, and 69 were more abundant under nitrate nutrition (Supplementary Dataset S1; Supplementary Fig. S2).

**Fig. 1.**
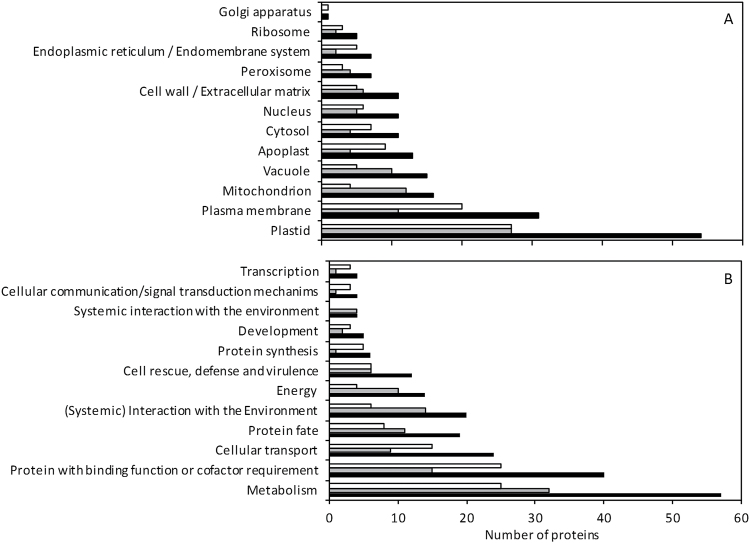
Classification of differentially expressed proteins into cellular components using TAIR/TIGR GO annotation (**A**) and into functional categories using the GO annotation in the MIPS-FunCat database (**B**). White bars represent proteins more abundant under nitrate nutrition; grey bars, proteins more abundant under ammonium nutrition; and black bars, all the differentially expressed proteins. The analysis was done using the BioMaps module of VirtualPlant 1.3 software.

In order to gain a more detailed description of the differentially expressed proteins we had identified, we used the BioMaps module of VirtualPlant 1.3 software (Katari *et al.*, 2010) to explore their distribution across gene ontology (GO) categories. Proteins were classified into cellular components using GO annotations of TAIR/TIGR and into functional categories using the GO annotations in the MIPS-FunCat (Ruepp *et al.*, 2004) (Fig. 1). With regard to cellular component classification, many of the differentially expressed proteins were associated with plastids, followed by those associated with the plasma membrane and the mitochondria (Fig. 1A). One could expect to find a predominant differential regulation of plastidic proteins because nitrite reduction takes place in this compartment; nevertheless, a similar number of proteins associated with plastids were found regardless of the nutrition type. Proteins associated with mitochondria or the vacuole mainly showed greater abundance under ammonium nutrition. By contrast, proteins classified within the cytosol, apoplast, or endoplasmic reticulum cellular components primarily showed increased content under nitrate nutrition (Fig. 1A). Therefore, these data suggest a differential cell compartment response for plants grown under different nitrogen sources.

Classification into functional categories showed that most of the differentially regulated proteins were associated with metabolism, with a similar proportion of proteins in both nutritional regimes falling into this category (Fig. 1B). The largest differences in protein expression found between treatments were in the categories of ‘transcription’, ‘cellular communication/signal transduction’, ‘protein synthesis’, ‘cellular transport’, and ‘protein with binding function’, in which proteins with higher expression under nitrate nutrition predominated. However, the categories ‘energy’ and ‘(systemic) interaction with the environment’ were predominantly composed of proteins whose expression was higher under ammonium nutrition (Fig. 1B).

**Fig. 2.**
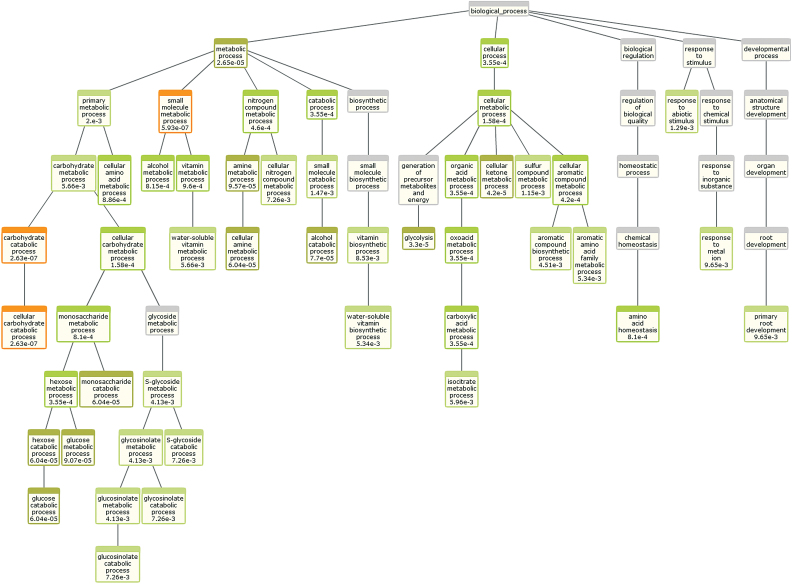
Biological process GO enrichment analysis of the proteins found with higher abundance under ammonium nutrition. The analysis was done using the BioMaps module of VirtualPlant 1.3 software. The *P* value corresponding to each term is indicated inside the diagram boxes (*P* < 0.01). This figure is available in colour at *JXB* online.

Overrepresentation of GO Biological Process was also analysed by the BioMaps module of VirtualPlant 1.3 (Katari *et al.*, 2010) using the *A. thaliana* TAIR 10 genome as a reference, Fisher’s exact test, and a *P* value cut-off of *P* ≤ 0.01. This analysis was performed for all the differentially expressed proteins together (Supplementary Fig. S3) and also separately for the proteins more abundantly expressed under nitrate (Supplementary Fig. S4) or ammonium nutrition (Fig. 2). When all 144 differentially expressed proteins were included, GO enrichment analysis highlighted the change in carbon and nitrogen metabolism (Supplementary Fig. S3). The analysis with the 69 proteins with higher content under nitrate nutrition showed that, overall, amino acid metabolism and, more precisely, lysine metabolism biological processes were significantly enriched (Supplementary Fig. S4). Finally, the results obtained by analysing the 75 proteins identified with a higher content under ammonium nutrition also revealed that amino acid and carbon metabolism and, interestingly, glucosinolate catabolic processes were enriched (Fig. 2). Myrosinase 1 (TGG1, At5g26000) and Myrosinase 2 (TGG2, At5g25980), essential enzymes in glucosinolate catabolism, were more abundant under ammonium nutrition, with 2.1- and 2.2-fold higher levels, respectively (Supplementary Dataset S1).

### Glucosinolate metabolism is modulated by the nitrogen source

**Fig. 3.**
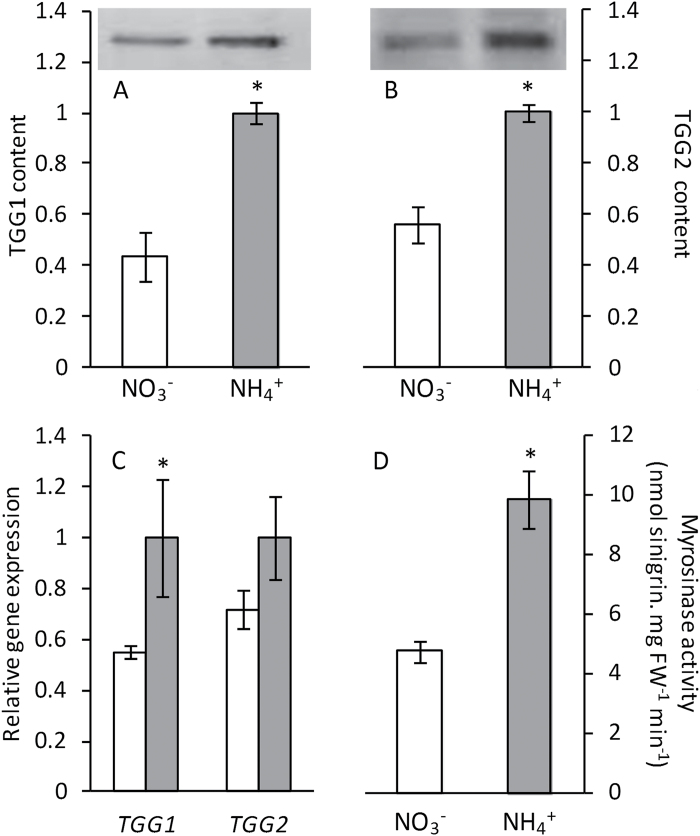
TGG1 (**A**) and TGG2 (**B**) content, and gene expression (**C**) and myrosinase activity (**D**) of *A*. *thaliana* plants grown with nitrate or ammonium as the nitrogen source. White bars represent plants grown under nitrate nutrition, and grey bars under ammonium nutrition. Values represent mean±SE (*n*=6). Statistical differences according to a Student’s t-test *P* value < 0.05 are indicated by an asterisk.

In order to complement and validate the iTRAQ-based LC-MS/MS analysis, western blotting assays were performed to check TGG1 and TGG2 levels. In agreement with iTRAQ results, TGG1 and TGG2 levels determined by western blotting were also higher under ammonium nutrition than nitrate nutrition. The densitometric quantification of the bands revealed very similar values to the ones obtained by proteomics for both TGG1 and TGG2 (Fig. 3A, B). Both *TGG1* and *TGG2* gene expression levels were also higher under ammonium nutrition, most notably *TGG1*, whose expression was twice what it was under nitrate nutrition (Fig. 3C). Furthermore, myrosinase activity values in plants grown under ammonium nutrition were twice those observed in nitrate-fed plants (Fig. 3D).

**Fig. 4.**
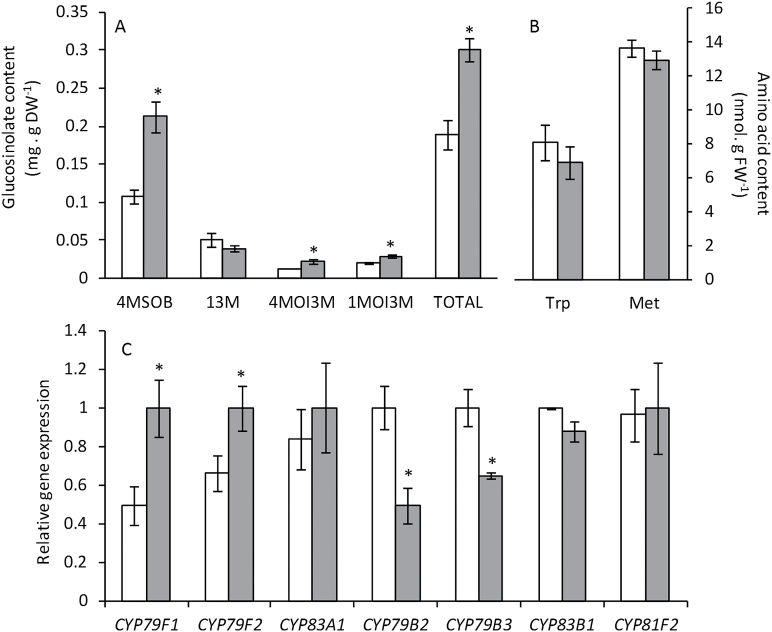
Glucosinolates content (**A**), Met and Trp content (**B**), and glucosinolate biosynthesis gene expression (**C**) in *A*. *thaliana* plants grown with nitrate or ammonium as the nitrogen source. White bars represent plants grown under nitrate nutrition, and grey bars under ammonium nutrition. Values represent mean±SE (*n* = 6). Statistical differences according to a Student’s t-test *P* value < 0.05 are indicated by an asterisk.

To further investigate the glucosinolate metabolic pathway, we determined glucosinolate content by LC-MS. Ten different glucosinolates were detected in Arabidopsis leaves (Supplementary Table S1) but their accumulation levels allowed us to quantify only four of them (Fig. 4A). Of the four glucosinolates quantified, glucoraphanin (4MSOB, 4-methylsulfinylbutyl), 4-methoxyglucobrassicin (4MO3IM, 4-methoxyindol-3-ylmethyl), and neoglucobrassicin (1MOI3M, 1-methoxyindol-3-ylmethyl) accumulated under ammonium nutrition, whereas the level of glucobrassicin (I3M, indol-3-ylmethyl) was similar for both nutrition regimes (Fig. 4A). Glucoraphanin is an aliphatic glucosinolate derived from (homo)methionine whereas glucobrassicin and its 4-methoxy and 1-methoxy derivatives are indolic glucosinolates derived from tryptophan. Although there was evidence of glucosinolate accumulation, both methionine and tryptophan levels were similar between the two nutrition regimes (Fig. 4B). The expression of several cytochrome P450 genes involved in the glucosinolate biosynthetic pathway was also modulated by the nitrogen source. *CYP79F1* and *CYP79F2* from the aliphatic glucosinolate pathway showed greater expression levels under ammonium nutrition whereas *CYP83A1* expression was not altered. In contrast, the expression of both *CYP79B2* and *CYP79B3*, from the indolic glucosinolate pathway, was higher under nitrate nutrition whereas *CYP83B1* and *CYP81F2* expression was not affected by the nitrogen source (Fig. 4C).

### Broccoli plants under ammonium nutrition also revealed glucosinolate accumulation and myrosinase activation

**Fig. 5.**
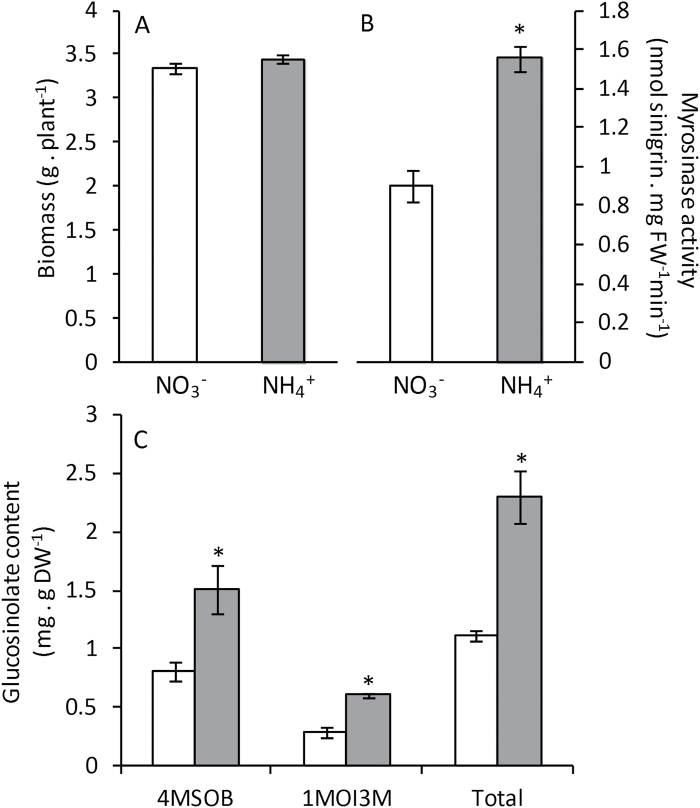
Biomass (**A**), myrosinase activity (**B**), and glucosinolate content (**C**) in broccoli plants grown with nitrate or ammonium as the nitrogen source. White bars represent plants grown under nitrate nutrition, and grey bars under ammonium nutrition. Values represent mean±SE (*n*=6). Statistical differences according to a Student’s t-test *P* value < 0.05 are indicated by an asterisk.

To investigate whether the response observed in the model plant *A*. *thaliana* grown under axenic hydroponic conditions also occurs in an important Brassica crop species, we grew broccoli plants (cv. Monaco) in perlite and vermiculite under exclusively nitrate or ammonium nutrition at an N concentration of 5mM. This concentration was chosen to achieve a very mild ammonium stress similar to that experienced by Arabidopsis, in which plant biomass was not affected, while sufficient N was supplied for proper plant growth. Regarding plant biomass, as observed for Arabidopsis, plants grown at this ammonium concentration did not show signs of toxicity because the biomass accumulation was nearly equivalent when comparing both nutrition regimes (Fig. 5A). Interestingly, myrosinase activity was around 60% higher under ammonium nutrition (Fig. 5B). As observed in Arabidopsis, broccoli plants also accumulated glucosinolates (Fig. 5C). Thus, these data confirm that ammonium nutrition alters glucosinolate metabolism in not only Arabidopsis but also broccoli.

## Discussion

Most plants display symptoms of toxicity when grown under ammonium-based nutrition. However, the external ammonium concentration threshold beyond the appearance of these symptoms greatly depends both on the genotype and on environmental conditions. Some of the most commonly used markers of ammonium stress are plant biomass, chlorophyll content, and ammonium accumulation. None of these parameters varied in the present study but other signs of ammonium-induced stress were evident, including anthocyanin accumulation, which has also been observed in Arabidopsis exclusively supplied with ammonium as its source of N (Helali *et al.*, 2010). Controlling the pH of the external medium has proved to be essential for ammonium tolerance (Chaillou *et al.*, 1991; Sarasketa *et al.*, 2016), and although Arabidopsis has been described as being very sensitive to ammonium nutrition, in this work we managed to induce a very mild degree of ammonium stress by buffering the nutrient solution and maintaining the external medium pH above 6 throughout the study.

Ammonium nutrition is generally treated as a stressful situation. Nevertheless, it has been observed that the plant’s metabolic adaptation to this nitrogen source may also have positive effects on its performance, even protecting the plant from other stresses. For example, ammonium-tolerant plants have also demonstrated tolerance to stresses such as soil acidity (Britto and Kronzucker, 2002). Similarly, there is also evidence that ammonium nutrition improves the response of some species exposed to high concentrations of atmospheric CO_2_ (Bloom *et al.*, 2010) and enhances plant tolerance to salinity or drought (Gao *et al.*, 2010). Moreover, plants cultured with ammonium have sometimes been shown to be better prepared to face pathogen attacks, such as tomato plants (*Solanum lycopersicum*) that were more resistant to virulent *Pseudomonas* infection (Fernández-Crespo *et al.*, 2015). In terms of food quality, a frequent characteristic associated with ammonium-fed plants is an increase in protein content, which is commonly associated with the need to increase NH_4_^+^ assimilation to prevent its accumulation. For example, this side effect of ammonium nutrition has been shown to increase the level of reserve proteins in wheat grain (*Triticum aestivum*) while enhancing its bread-making qualities (Fuertes-Mendizabal *et al.*, 2013). In the present work, ammonium nutrition led to an increase in glucosinolate content and the activation of enzymes responsible for their degradation, myrosinases, in both Arabidopsis and broccoli. The Arabidopsis genome encodes for six classical myrosinases (TGG1–6), of which TGG1 and TGG2 are the most highly expressed and abundant; indeed, the double mutant *tgg1tgg2* is almost completely impaired in glucosinolate breakdown (Barth and Jander, 2006). Interestingly, both TGG1 and TGG2 content and expression were more abundant under ammonium nutrition, accompanied by an increase in total myrosinase activity. In Arabidopsis, the observed increase in glucosinolate content correlated with expression of *CYP79F1* and *CYP79F2*, which encode enzymes of aliphatic glucosinolate biosynthesis. In regard to indolic glucosinolates, it is difficult to explain the observed lower expression of *CYP79B2* and *CYP79B3* under ammonium nutrition while two of the three quantified indolic glucosinolates had higher expression under this nutrition regime. In this sense, further work will be needed to evaluate the post-translational regulation of these P450s, together with an in-depth analysis of the whole range of indolic glucosinolates.

Different functions have been proposed for both glucosinolates and their degradation products. The most widely known function of glucosinolates is based on the toxicity of their degradation products for herbivores, but their ability to protect plants from biotrophic pathogen infections has also been described (Bednarek *et al.*, 2009). In addition, glucosinolates are derived from amino acids and contain nitrogen and sulfur; thus, they are closely related to primary metabolism, and whenever nitrogen and sulfur metabolism are affected, a subsequent change in glucosinolate metabolism is commonly reported. Indeed, one of the suggested functions of glucosinolates is as sulfur-storage compounds, demonstrated by the fact that their degradation is promoted when there is a sulfur deficiency (Falk *et al.*, 2007.). In the present study, the nutrient solution of ammonium-fed plants had more available sulfate compared to nitrate-fed plants because the ammonium was supplied as 1mM (NH_4_)_2_SO_4_. However, the observed myrosinase activation in ammonium-fed plants is contrary to the proposed glucosinolate degradation under conditions of sulfur deficiency. Nevertheless, we conducted a complementary experiment to rule out the possibility that an alteration in glucosinolate metabolism could be related to sulfur imbalance. This involved growing plants with equal amount of sulfate under both nitrogen treatments, providing extra sulfate to nitrate-fed plants, and by the use of 2mM NH_4_Cl as an alternative ammonium source; we again observed myrosinase activation in the presence of ammonium (Supplementary Fig. S1). Moreover, broccoli experiments were performed with NH_4_Cl as the ammonium source and, as reported for Arabidopsis, we observed both glucosinolate accumulation and myrosinase activation (Fig. 5). Therefore, glucosinolate metabolism alteration seems to be specific to the ammonium supply.

Nitrogen availability has also been shown to be crucial for glucosinolate synthesis; excessive N fertilization may cause glucosinolates to accumulate, and a low N supply could cause glucosinolate content to decrease (Yan and Chen, 2007; Omirou *et al.*, 2009; He *et al.*, 2014). One of the strategies used by plants when facing ammonium stress is to increase ammonium assimilation to prevent it accumulating to toxic levels. Thus, one hypothesis could be that plants direct ammonium ions towards the glucosinolate pathway as part of a metabolic strategy to prevent toxic accumulation of NH_4_^+^. Further to this, another strategy reported for ammonium stress tolerance is ammonium compartmentalization into vacuoles (Wells and Miller, 2000; Loqué *et al.*, 2005). Interestingly, in the present study, 12 of the 16 differentially expressed proteins associated with the vacuole were found to have higher expression under ammonium nutrition than nitrate nutrition. Glucosinolates are mainly stored in the vacuoles, as well as in the xylem and apoplast (Jørgensen *et al.*, 2015). Myrosinase enzymes are also usually found in vacuoles, thus underlining the importance of this compartment in the cell’s overall response to ammonium stress (Shirakawa *et al.*, 2014). However, glucosinolates and myrosinases are thought to be located in different cell types and their *in vivo* interaction is still not completely understood. The general model states that glucosinolates and myrosinases are physically separate and when attacked by pests both components are exposed together, leading to glucosinolate hydrolysis (Wittstock and Burow, 2010). However, this mechanism does not explain how myrosinase is activated to degrade glucosinolates in intact plants under certain abiotic conditions, for instance when experiencing sulfur deficiency (Maruyama-Nakashita *et al.*, 2003; Falk *et al.*, 2007) or, as in the present study, upon ammonium provision. Thus, the possibility that myrosinase is located as an ‘inactive form’ within the same subcellular localization as glucosinolates still cannot be completely ruled out (Kissen *et al.*, 2009). Finally, glucosinolate transport between cells, both by specific transporters or across plasmodesmata, also seems to be crucial for their function (Madsen *et al.*, 2014; Jørgensen *et al.*, 2015).

Apart from mineral nutrition, other environmental factors may also affect glucosinolate content, such as salinity (López-Berenguer *et al.*, 2008), light (Huseby *et al.*, 2013), and elevated CO_2_ (Schonhof *et al.*, 2007), and glucosinolate breakdown has been proposed to play a role in cellular signalling response to abiotic stress. For instance, exogenous glucosinolate provision mimicked the effect of abscisic acid on stomatal opening in a TGG1-dependent manner (Zhao *et al.*, 2008). Similarly, the absence of aliphatic glucosinolates had an impact on Arabidopsis exposure upon salt stress (Martínez-Ballesta *et al.*, 2015). Furthermore, the balance of other hormones important for plant responses upon environmental alterations, such as jasmonic acid and salicylic acid, seem to be related to glucosinolate metabolism regulation (Schreiner *et al.*, 2011; Guo *et al.*, 2013b). Thus, glucosinolates appear to be active actors in plant response to abiotic stress but the mechanisms underlying the role of both glucosinolates and their degradation products under abiotic stresses still need to be deciphered; at present, no targets have been identified. The use of mutants altered in different steps of the glucosinolate metabolic pathway, including biosynthesis and degradation, will be extremely helpful to elucidate the role of these secondary metabolites under ammonium stress.

Pesticide use entails not only an environmental hazard but also a human health risk, with many studies commonly reporting detectable, or even quantifiable, amounts of these chemicals in edible plant products (Nougadère *et al.*, 2011; Bonnechère *et al.*, 2012); hence, consumers and breeders welcome alternative strategies for pest control. Therefore, modifying plant nutrition to foster their defensive capacity, for instance by taking advantage of the properties of glucosinolates, is of particular interest. Further to this, glucosinolates have also been associated with health-promoting activities. In particular, sulforaphane, which is produced from glucoraphanin hydrolysis, the main glucosinolate accumulated in the present work under ammonium nutrition, is thought to contribute to a reduction in the risk of carcinogenesis and heart disease when consumed as part of the human diet (Traka and Mithen 2011; Houghton *et al.*, 2013). Indeed, the selection of varieties with high glucoraphanin content is an important area of research (Traka *et al.*, 2013). Thus, the results presented here open a promising avenue for Brassicaceae culture to improve both their defensive capacity and nutritional value by controlling the nitrogen source.

## Supplementary data

Supplementary data are available at *JXB* online.

Figure S1. Myrosinase activity of *Arabidopsis thaliana* plants grown under different nitrogen sources: 1mM (NH_4_)_2_SO_4_, 1mM Ca(NO_3_)_2_ + 1mM CaSO_4_, and 2mM NH_4_Cl.

Figure S2. Volcano plots representing the fold-change of identified proteins with associated *P* values from the pair-wise quantitative comparisons of plants grown under nitrate or ammonium nutrition.

Figure S3. Biological process GO enrichment analysis of all the differentially expressed proteins with respect to the N source.

Figure S4. Biological process GO enrichment analysis of the differentially expressed proteins found with higher abundance under nitrate nutrition.

Table S1. Identification and quantification of glucosinolates by LC-MS in leaves of Arabidopsis plants grown under nitrate or ammonium nutrition.

Dataset S1. List of proteins identified, quantified, and differentially expressed between Arabidopsis plants grown under nitrate or ammonium nutrition.

Dataset S2. List of all peptides identified.

Dataset S3. List of proteins associated to the enriched GO terms.

Supplementary Data
