# Supplementary material for: Quantitative proteomics reveals the importance of nitrogen source to control glucosinolate metabolism in Arabidopsis thaliana and Brassica oleracea
Source: J Exp Bot. 2016 Apr 16;67(11):3313–23. doi: 10.1093/jxb/erw147 (PMC4892723; doi:10.1093/jxb/erw147)
Supplement: Supplementary Data [file supp_67_11_3313__index.html]

Quantitative proteomics reveals the importance of nitrogen source to control glucosinolate metabolism in Arabidopsis thaliana and Brassica oleracea — Quantitative proteomics reveals the importance of nitrogen source to control glucosinolate metabolism in Arabidopsis thaliana and Brassica oleracea — Supplementary Data 

# Quantitative proteomics reveals the importance of nitrogen source to control glucosinolate metabolism in *Arabidopsis thaliana* and *Brassica oleracea*

## Supplementary Data

Data files

- supplementary\_dataset\_S1.xlsx - Supplementary Data
- supplementary\_dataset\_S2.xlsx - Supplementary Data
- supplementary\_dataset\_S3.xlsx - Supplementary Data
- supplementary\_table\_S1\_figures\_S1\_S4.pdf - Supplementary Data
