# Supplementary material for: Quantitative proteomics reveals the importance of nitrogen source to control glucosinolate metabolism in Arabidopsis thaliana and Brassica oleracea
Source: J Exp Bot. 2016 Apr 16;67(11):3313–23. doi: 10.1093/jxb/erw147 (PMC4892723; doi:10.1093/jxb/erw147)

Table S1. Identification and quantification of glucosinolates by LC-MS in leaves of Arabidopsis plants grown under nitrate or ammonium nutrition.

Systematic name	Common name	Content (mg . g FW	
		Nitrate	Ammonium
3-methylsulfinylpropyl – GLS	Glucoiberin	n.q.	n.q.
4-methylsulfinylbutyl – GLS	Glucoraphanin	0.107 ± 0.01	0.212 ± 0.02
5-methylsulfinilpentyl – GLS	Glucoalyssin	n.q.	n.q.
6-methylsulfinylhexyl – GLS	Glucohesperin	n.q.	n.q.
7-methylsulfinylheptyl – GLS	Glucosibarin	n.q.	n.q.
4-methylthyobutyl – GLS	Glucoerucin	n.q.	n.q.
8-methylsulfinyloctyl – GLS	Glucohirsutin	n.q.	n.q.
3-indolylmethyl – GLS	Glucobrassicin	0.051 ± 0.01	0.039 ± 0.00
4-methoxy-3-indolylmethyl – GLS	Methoxyglucobrassicin	0.013 ± 0.00	0.023 ± 0.00
1-methoxy-3-indolylmethyl – GLS	Neoglucobrassicin	0.020 ± 0.00	0.029 ± 0.00

Figures S1. Myrosinase activity of *Arabidopsis thaliana* plants grown under different nitrogen sources: 1 mM $(NH_4)_2SO_4$, 1 mM $Ca(NO_3)_2$ + 1 mM $CaSO_4$, and 2 mM NH_4Cl. Values represent mean ± se (n = 3). Statistical differences according to a Student's t-test P-value ≤ 0.05 are indicated by an asterisk.

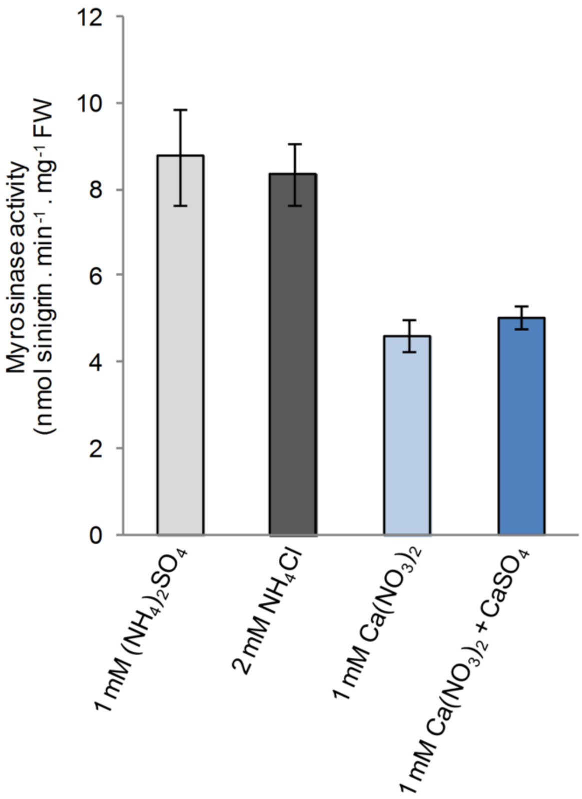

Figure S2. Volcano plot representing the fold-change of identified proteins with associated *P* values from the pair-wise quantitative comparisons of plants grown under nitrate or ammonium nutrition. In green, very significantly changed proteins (*P* < 0.01), in blue, significantly changed proteins (*P* < 0.05) and in red, unchanged proteins between the treatments..

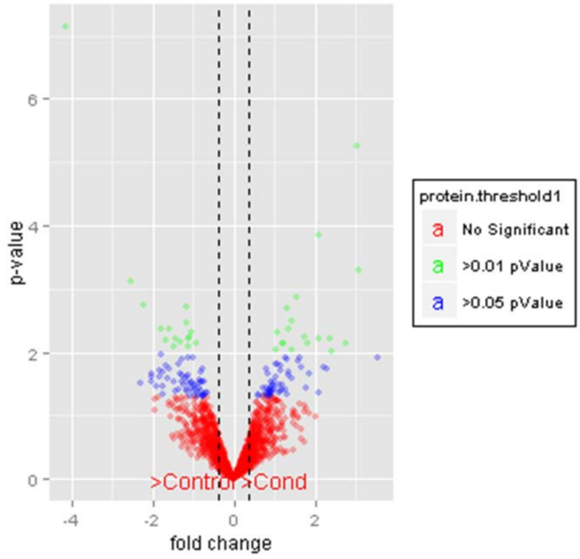

Figure S3. Biological process GO enrichment analysis of all the differentially expressed proteins with respect to the N source. The analysis was done using the BioMaps module of VirtualPlant 1.3 software. The p-value corresponding to each term is indicated inside the diagram boxes ($p<0.01$).

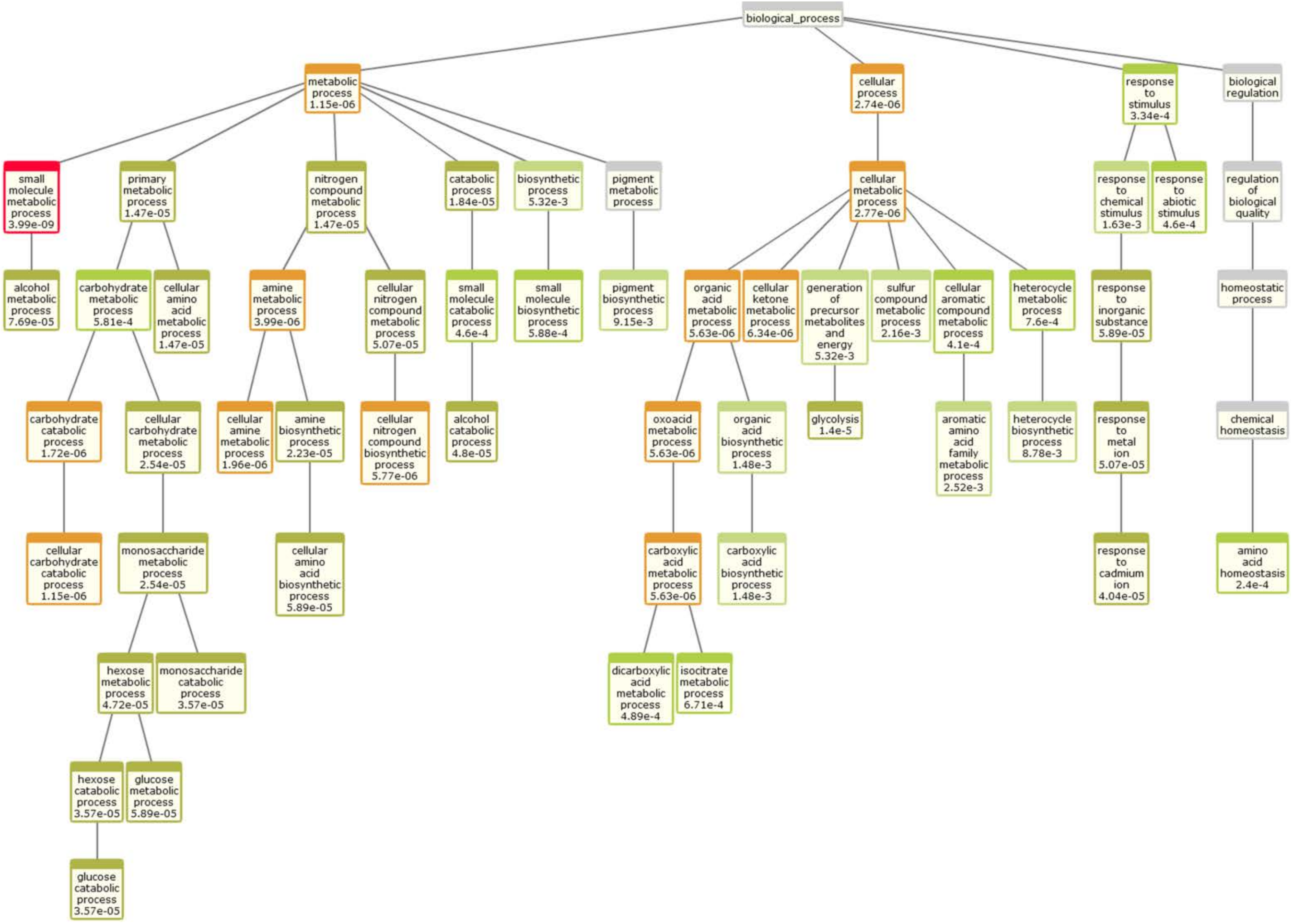

Figure S4. Biological process GO enrichment analysis of the differentially expressed proteins found with higher abundance under nitrate nutrition. The analysis was done using the BioMaps module of VirtualPlant 1.3 software. The p-value corresponding to each term is indicated inside the diagram boxes (p<0.01).

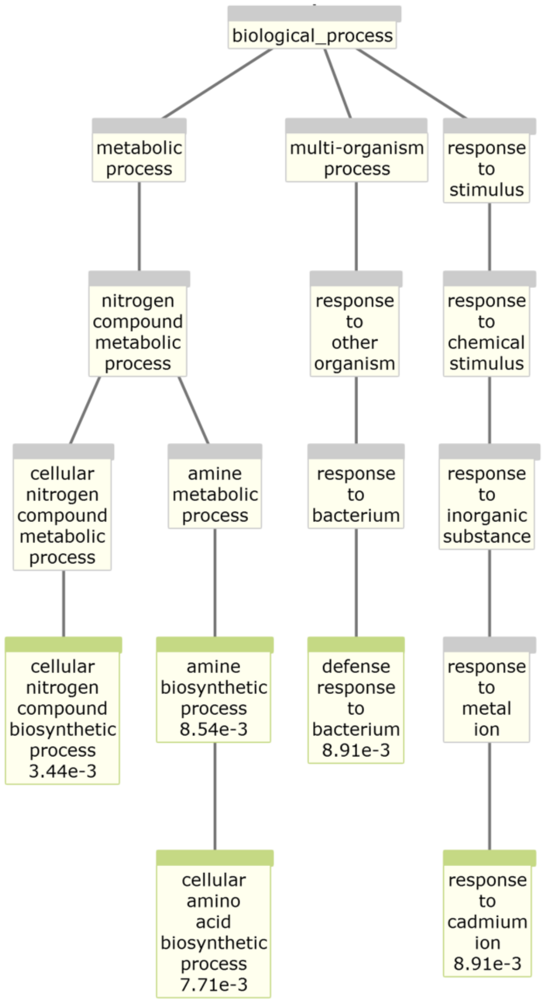

Supplement: Supplementary Data [file supp_erw147_supplementary_table_S1_figures_S1_S4.pdf]
